# Reproductive life after in vitro fertilization

**DOI:** 10.1172/JCI210577

**Published:** 2026-08-17

**Authors:** Serdar E. Bulun

Different assisted reproductive technologies (ARTs) are associated with varying levels of risks to offspring health. Risk seems to increase with the invasiveness of the technology (1). There has been a long-standing concern that in vitro fertilization (IVF), which involves maintaining and manipulating the gametes and embryos in culture outside the body for days, is associated with defective epigenetic programming leading to readily recognizable or subtle developmental abnormalities (2). The addition to IVF of intracytoplasmic sperm injection (ICSI), which bypasses another natural reproductive step, increases this risk (1). While most children conceived by ART are healthy, IVF with or without ICSI is associated with an increased risk of rare imprinting disorders (e.g., Beckwith-Wiedemann syndrome), and subtle epigenetic perturbations at less well-known loci (e.g., hypospadias or cardiovascular anomalies) may influence disease susceptibility later in life (1). Moreover, as discussed below, mouse studies suggest that some of these epigenetic perturbations may manifest in future generations.

Three articles recently published in the *JCI* and *JCI Insight* demonstrated transgenerational effects of IVF or IVF-ICSI on behavior or gonadal biology in mouse models. First-generation (F1) mice generated by IVF-ICSI exhibited behavioral abnormalities, which persisted in second-generation (F2) mice (3). F1 mice generated by IVF alone showed changes in testicle–to–body weight ratios, serum testosterone levels, testicular morphology, gene expression, and DNA methylation; changes in testicular DNA methylation persisted in F2 mice (4). In this issue, Rhon-Calderon and co-authors demonstrated that IVF-generated F1 female mice exhibited altered ovarian function, reduced follicle reserve, disrupted endocrine profiles, and transcriptomic and epigenetic changes in oocytes and granulosa cells consistent with premature reproductive decline. These ovarian changes became more prominent with advancing age. IVF-generated F1 females produced fewer F2 pups due to a higher resorption rate (5).

What do these observations in mouse models mean for human offspring born with the assistance of IVF? As noted above, IVF in humans has been associated with an increased risk of imprinting disorders in offspring, confirming that epigenetic perturbations do occur and can have clinical consequences (1). The effects of IVF-induced epigenetic alterations in reproductive or other tissue function, however, remain largely unknown. Robust human evidence for transgenerational inheritance through gametes remains limited, and no transgenerational human data are available in relation to ART or IVF. No human studies have directly assessed whether women conceived by IVF have diminished ovarian reserve or earlier menopause compared with naturally conceived women. Interestingly, women who have conceived children by IVF show accelerated epigenetic aging, although this probably reflects underlying infertility rather than a treatment effect (6).

The first generation of IVF-conceived women are now reaching their late 30s and 40s, the age at which diminished ovarian reserve and premature ovarian insufficiency would become clinically apparent. Currently, there is no clinical recommendation to screen IVF-conceived women differently for ovarian reserve, and the American Society for Reproductive Medicine notes that ovarian reserve testing should be interpreted in a clinical context and is not recommended as a screening tool for fertile women (7). The mouse data from Rhon-Calderon et al. raise an important hypothesis that warrants prospective human cohort studies (5).

## Conflict of interest

The author has declared that no conflict of interest exists.

## Version 1 08/17/2026

Electronic publication
